# Supplementary material for: Preparation and Tribological Properties of Bismaleimide Matrix Composites Reinforced with Covalent Organic Framework Coated Graphene Nanosheets
Source: Polymers (Basel). 2022 Aug 12;14(16):3289. doi: 10.3390/polym14163289 (PMC9416587; doi:10.3390/polym14163289)
Supplement: Supplementary file 1 [file polymers-14-03289-s001.zip › polymers-1850301-supplementary.pdf]

Electronic Supplementary Information

Preparation and Tribological Properties of Bismaleimide Matrix Composites Reinforced with Covalent Organic Framework Coated Graphene Nanosheets

Chao Liu[1,2*], Xin Xue[3], Qiming Yuan[3], Yang Lin[3], Yan Bao[2], Yinkun He[3] and Wenbo Zhang[1]

[1] *Key Laboratory of Auxiliary Chemistry and Technology for Chemical Industry, Ministry of Education, Shaanxi Collaborative Innovation Center of Industrial Auxiliary Chemistry and Technology, Shaanxi University of Science and Technology, 710021 Xi'an, China*

[2] *College of Bioresources Chemical and Materials Engineering, Shaanxi University of Science and Tech-nology, 710021 Xi'an, China*

[3] *College of Chemistry and Chemical Engineering, Shaanxi University of Science and Technology, 710021 Xi'an, China*

[*] *Correspondence: author: Shaanxi University of Science and Technology, 710021 Xi'an, PR China.; E-mail address: lc1010158@163.com. Phone: +86 15829552486.*

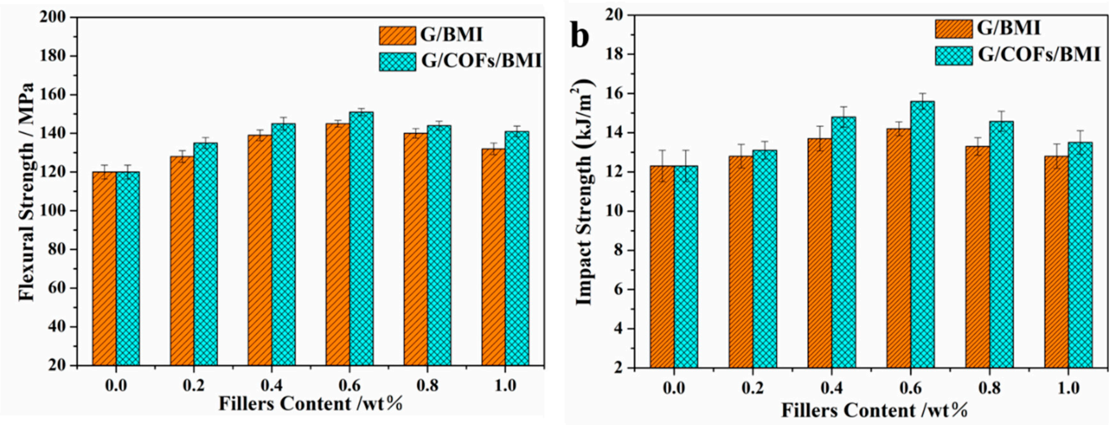

Figure S1. Relationship between mechanical properties and fillers content: flexural strength (a) and impact strength (b) of the composites, respectively.

When served in applications with high load and sliding velocity, it is of great importance for the composites to have outstanding flexural strength and impact strength to resist deformation and bending failure. In this study, the dependency of the flexural strength and impact strength of the G/COFs/BMI composites on the content of the G/COFs is shown in Figure S1.

It can be seen that the composites containing the G/COFs exhibit higher strength values than those of the G at almost all the filler content (See Figure S1 a, b). As shown that the flexural strength and impact strength of composites fall at first, then rise as the number of fillers increases. When the filler addition is 0.6 wt%, the flexural strength and impact strength of G/COFs/BMI composite reach the maxima with 151 MPa and 15.6 kJ/m^2. Compared to neat BMI (120 MPa and 12.3 kJ/m^2), it is improved by 25.8% and 26.8%, respectively. This phenomenon is attributed to the unique sheets structure of hybrid graphene and the interfacial adhesion between G/COFs and BMI matrix, which can greatly improve the flexural strength and impact strength of BMI resin[1].

However, when the addition amount is more than 0.6 wt%, the flexural strength and impact strength of G/COFs/BMI composites decrease. But they are still higher than those of neat BMI. It may be because excessive fillers can not be well dispersed in the BMI matrix and agglomerate to cluster [2]. Consequently, the advantages of the fillers can not be fully utilized and the flexural strength and impact strength of the composites decreases with the uneven distribution of the fillers in the BMI matrix [3].

Reference:

[1] Chan, J.X.; Wong, J.F.; Petrů, M.; Hassan, A.; Nirmal, U.; Othman, N.; Ahmad Ilyas, R. Effect of nanofillers on tribological properties of polymer nanocomposites: A review on recent development. *Polymers.* ***2021****, 13,* 2867.

[2] Liu, C.; Dong, Y.F.; Lin, Y.; Yan, H.X.; Zhang, W.B.; Yan, B.; Ma, J.Z. Enhanced mechanical and tribological properties of gra-phene/bismaleimide composites by using reduced graphene oxide with non-covalent functionalization. *Compos. Part B Eng.* ***2019****, 165,* 491-499.

[3] Zhang, Y.B; Yan H.X; Xu, P.L.; Guo, L.L; Yang, K.M.; Rui Liu, R.; Feng, W.X. A novel POSS-containing polyimide: Synthesis and its composite coating with graphene-like MoS_2 for outstanding tribological performance. *Prog. Org. Coat.* ***2021****, 151:* 106013.
